# Association of body mass index with clinical outcome of primary WHO grade 4 glioma

**DOI:** 10.3389/fonc.2024.1318785

**Published:** 2024-04-29

**Authors:** Jiejun Wang, Zhaonian Hao, Ruyuan Li, Weiqi Wu, Na Huang, Kangna Zhang, Shuyu Hao, Jie Feng, Junsheng Chu, Nan Ji

**Affiliations:** ^1^Department of Neurosurgery, Beijing Tiantan Hospital, Capital Medical University, Beijing, China; ^2^National Cancer Center, Chinese Academy of Medical Sciences and Peking Union Medical College, Beijing, China; ^3^Beijing Neurosurgical Institute, Capital Medical University, Beijing, China

**Keywords:** glioma, glioblastoma, body mass index, obesity paradox, overall survival

## Abstract

**Background:**

The prognostic value of body mass index (BMI) in primary WHO grade 4 gliomas is not widely acknowledged. This study aims to assess the survival outcomes of patients with different BMIs.

**Methods:**

Real-world data of patients diagnosed with primary WHO grade 4 (2021 version) glioma was assessed. All 127 patients admitted in this study were administered with standard-of-care from September 2018 to September 2021. The outcomes of overall survival and progression-free survival were analyzed.

**Results:**

The baseline characteristics of clinical features, molecular features, and secondary treatment in BMI subsets showed no significant difference. The survival analyses showed a significantly superior overall survival (OS) in the overweight group compared to the normal weight group. A trend of better OS in the overweight group compared to the obesity group was observed. The univariate Cox regression demonstrated patients of round-BMI 25 and 26 had superior OS outcomes.

**Conclusion:**

In this real-world setting, patients with a BMI between 24 and 28 have superior overall survival. Patients in the proper BMI range may acquire survival benefits undergoing standard-of-care of primary WHO grade 4 gliomas. The prospective studies on a larger scale on these subsets of patients are necessary to solve the paradox of BMI in glioma.

## Introduction

Glioblastoma (GBM) is the most aggressive and common primary brain tumor with a poor prognosis (1, 2). The current standard-of-care (maximal safe resection-gross total resection, concurrent chemoradiotherapy, and adjuvant chemotherapy) results in a median survival time of merely 13.2 to 16.8 months (3). Despite ongoing efforts, the identification of subpopulations of glioblastoma using molecular markers has advanced further than the much-needed studies of developing novel therapeutic strategies, including isocitrate dehydrogenase (IDH) and O6-methylguanine (O6-MeG)-DNA methyltransferase (MGMT) promoter methylation status (4, 5). However, more high-power clinical prognostic biomarkers are necessary to be developed. The clinical characteristics such as age, gender, pathological type, lifestyle, surgical resections, and cognitive impairments showed capability in predicting clinical outcomes of glioma (6, 7).

BMI, generally used to distinguish different body obesity conditions, has been proposed as a feasible prognostic factor in predicting the clinical outcome of patients with various diseases. A substantial increment in the risk of various diseases has also been observed in underweight people (8, 9). As an easily obtained and simply used indicator in multiple clinical and research scenarios, BMI has been associated with tumor progression and prognosis in 17 of 22 cancers, including liver, colon, and postmenopausal breast cancer (10). Higher BMI has also been associated with lower mortality risk in patients undergoing surgical procedures. The obese population, defined by BMI, has been associated with better prognoses of cancer patients undergoing surgery, including rectal cancer, non-small-cell lung cancer, intra-abdominal cancer, hepatocellular carcinoma, and pancreatic cancer, and this is called the “obesity paradox” (11). The elevated BMI may be associated with better survival in patients with newly diagnosed GBM (12–14). However, a large prospective multicenter study found no relationship between BMI and survival in newly diagnosed and previously untreated GBM patients (15). Still, no consensus has been reached yet on the topic of obesity-paradox in GBM patients. In this real-world study, we aimed to explore the relationship between BMI with the overall survival (OS) and progression-free survival (PFS) of patients with primary WHO grade 4 glioma.

## Materials and methods

The ethical committee of Beijing Tiantan Hospital (JS2012-001-03) approved this cohort study. The study was performed in accordance with the Declaration of Helsinki, and conducted a retrospective review of medical records, with minimal risk to patients. All patients selected for this study gave their written consent.

### Study design and participants

**Figure 1:**
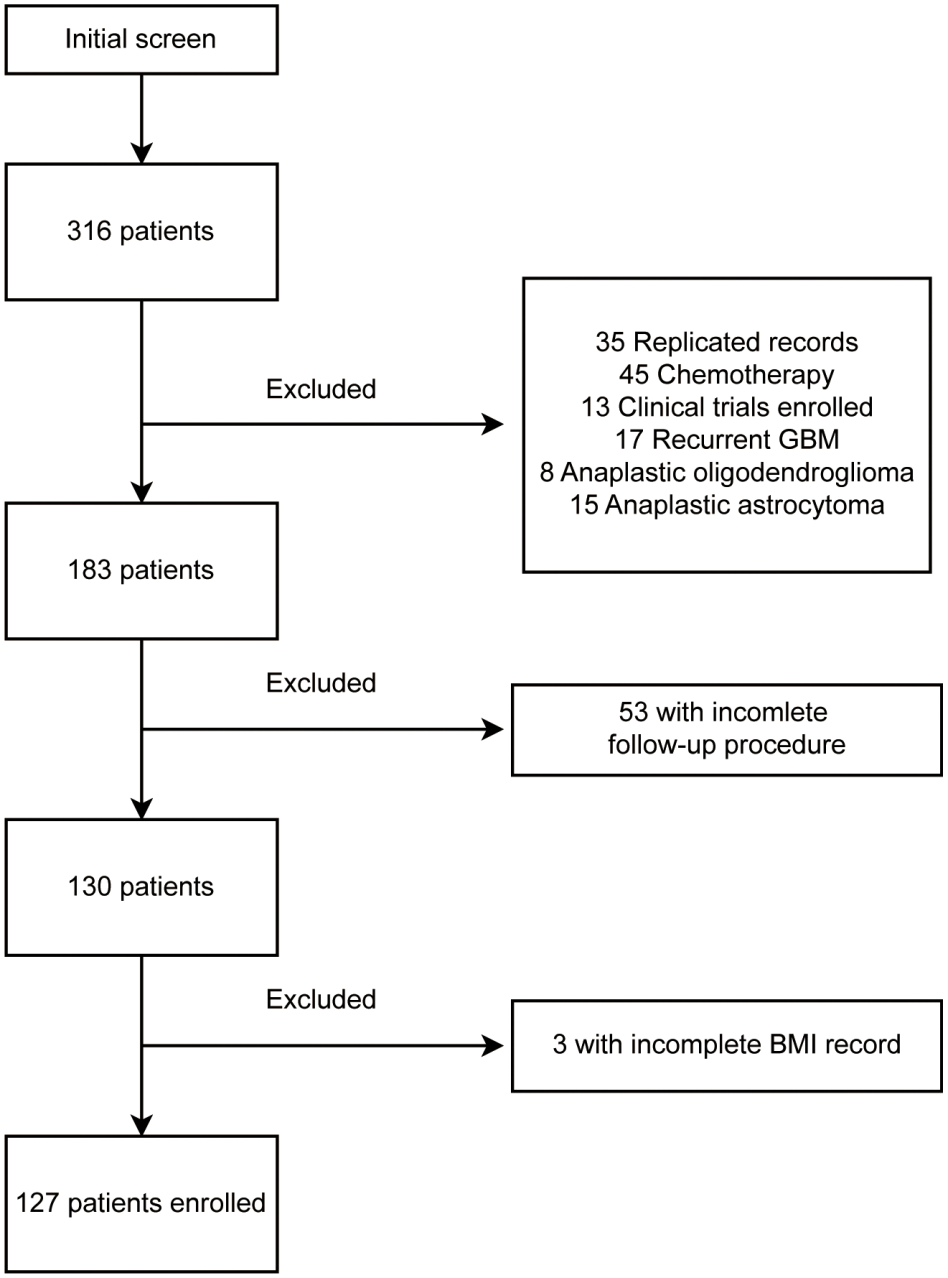
Flowchart of patient enrollment and exclusion.

This is a retrospective study of primary WHO grade 4 gliomas performed in Beijing Tiantan Hospital. A total of 316 patients, diagnosed with “glioblastoma” (WHO 2016 version), were recorded from September 2018 to September 2021. The follow-up procedure ended in September 2022. Of all excluded records shown in **Figure 1**, 35 were duplicated records from multiple hospitalizations, 45 were patients admitted specifically for chemotherapy, 13 were patients enrolled in interventional clinical trials, 17 were confirmed as recurrent glioblastomas, and 23 were patients diagnosed as anaplastic oligodendroglioma or anaplastic astrocytoma after surgery. We further excluded 53 patients without detailed follow-up data and three patients without weight and height records. BMI was calculated by the formula (BMI = weight in kilograms/(height in meters)^2^), the pre-surgery BMI was used in this study. Eventually, 127 patients were enrolled in this study. All patients were classified by their BMI into four groups: underweight (BMI, <18.5), normal weight (BMI, from 18.5 to 23.9), overweight (BMI, from 24 to 27.9), and obese (BMI, >=28).

### Data collection

Demographic, imaging data, molecular information, surgery, chemoradiotherapy, and follow-up records were collected from the electronic medical records system, and data were recorded into a standardized predesigned database. A team of trained clinicians performed the data processing and analyses. All data acquired from the electronic records were cross-checked by two clinicians.

### Interpretation of imaging information

All imaging results were reviewed and interpreted by experienced clinicians. Features such as tumor laterality, location, presence of contrast enhancement, and extent of resection were collected. Magnetic resonance images (MRI) before and within 72 h after operation were compared, and the extent of resection was divided into gross total resection (GTR, no postoperative evidence of residual tumor) and subtotal resection (STR, postoperative evidence of residual tumor).

### Histological and molecular characteristics

Histopathological records and molecular assays of glioma biomarkers were extracted, including IDH mutation status, MGMT promoter methylation status, telomerase reverse transcriptase (TERT) promoter mutation, and chromosome 1p19q co-deletion status. Mutational molecular characteristics were obtained via next-generation sequencing (NGS). The MGMT promoter methylation status was detected by pyrosequencing. The fluorescence *in situ* hybridization (FISH) was performed to delineate 1p19q co-deletion status. All enrolled patients were classified into GBM, GBM-NOS, and astrocytoma WHO grade 4, according to the 2021 version of WHO criteria. Tumors with pathologically microvascular proliferation or necrosis and essential molecular characteristics (IDH-wildtype) were classified as GBM; tumors with histopathological features of GBM but without essential molecular characteristics (IDH-wildtype) were classified as astrocytoma WHO grade 4; tumors with histopathological features of GBM but without available molecular information were classified as not otherwise specified (NOS) GBM.

### Definition of clinical characteristics

Patients’ clinical characteristics were recorded per the following definitions. History of epilepsy was defined as any type of epilepsy before surgical intervention of the brain tumor. History of anti-epilepsy drugs was defined as any usage of such agents before surgical intervention. Enzyme-inducing anti-epilepsy drugs (EIAED) were defined as anti-epileptic agents that induce the activity of hepatic mixed function oxidase enzymes, which interfere with the kinetics of other drugs. History of heart disease was defined as any type of heart disease including both vascular and non-vascular. History of hypertension was defined as blood pressure above 140/90 (mmHg) and clinically diagnosed in a medical institution. History of diabetes was defined as the formal diagnosis of diabetes before neurosurgery. Trauma history was defined as any type of trauma (not limited to cerebral trauma). The family history of malignancy was defined as any type of malignancy, excluding benign tumors. The history of cerebrovascular disease was defined as both hemorrhagic and ischemic diseases. The location of the brain lesion was categorized by tumor location as left, right, or bilateral.

### Outcomes and definitions

In this study, the OS and PFS of patients were set as primary and secondary outcomes, respectively. OS is defined as the date from initial pathologic diagnosis to the date of death or the date of last follow-up, and PFS is defined as the date from initial pathologic diagnosis to the date of progression assessed by MRI or the date of last follow-up.

### Tumor volume estimation

A gross-resection style of surgery was performed for patients with high precedence when possible. We therefore obtained tumor samples with relatively high integrity, which is fundamental for the veracity of recorded tumor diameters. All resected samples were measured promptly after surgery. Tumor volume (TV) was estimated using the formula: TV= α (length) × β (width) × γ (height) × 6/π.

### Treatment strategy

All patients underwent surgical resection, followed by conventional radiation therapy administrated at 2 Gy per fraction five days a week up to a total of approximately 60 Gy; patients additionally were administered daily temozolomide (75 mg/m^2^ orally) followed by standard maintenance temozolomide chemotherapy (150-200 mg/m^2^/d for 5 days every 28 days, 6 cycles), according to the protocol established by the European Organization for Research and Treatment of Cancer (EORTC) Brain Tumor and Radiotherapy Groups and the National Cancer Institute of Canada (NCIC) Clinical Trials Group.

### Follow-up

All patients were instructed to perform MRI at three-month intervals to follow up on the progression of lesions before discharging. Researchers collected clinical information every three months by telephone calls, including clinical symptoms, quality of life, cognitive status, and any adverse events associated with therapy. If we were unable to successfully contact a patient after two attempts, they were considered lost to follow-up, and their data were excluded from our analysis. If patients exhibited clinical progression, an MRI was conducted within two weeks. All MRIs were reviewed by two experienced clinicians.

### Statistical analysis

Statistical analyses were performed using SPSS 25.0 (IBM Corp., Armonk, NY, United States). Continuous and categorical variables were presented as median (IQR) and n (%), respectively. ANOVA analysis, χ² test, or Fisher’s exact test were performed in comparison of differences between groups where appropriate. The log-rank test was used in the comparison of OS and PFS between groups, which was plotted using the Kaplan–Meier method. The univariable Cox regression model was performed for hazard ratio (HR) estimation between groups. Statistical significance for the analyses was defined as P < 0.05. Missing values of individual records were excluded for its specific analysis.

## Results

Detailed demographic and clinical characteristics of patients within different BMI groups are shown in **Table 1**. No significant differences were found among BMI subsets for clinical features of sex, age, epilepsy history, usage of the anti-epilepsy drug, usage of enzyme-inducing anti-epilepsy drug (EIAED), heart disease history, hypertension, diabetes, trauma history, family history of malignancy, history of cerebrovascular disease, number of lesion(s), location of lesion(s), tumor volume, excision extension, IDH 1/2 mutation, MGMT promoter methylation, TERT promoter mutations, and integrated diagnosis.

**Table 1:** Characteristics of the patients enrolled in the study.

Characteristics	Healthy Weight(N=56)	Overweight(N=49)	Obesity(N=17)	Underweight(N=5)	Total(N=127)	^*^ P value
Sex						0.06
Female	21(16.54%)	20(15.75%)	8(6.30%)	5(3.94%)	54(42.52%)	
Male	35(27.56%)	29(22.83%)	9(7.09%)	0(0%)	73(57.48%)	
Age (Years)						0.80
Median[min-max]	57.00[29.00,75.00]	55.00[31.00,74.00]	53.00[29.00,79.00]	49.00[34.00,74.00]	55.00[29.00,79.00]	
Epilepsy						0.06
No	42(33.60%)	30(24.00%)	15(12.00%)	2(1.60%)	89(71.20%)	
Yes	13(10.40%)	18(14.40%)	2(1.60%)	3(2.40%)	36(28.80%)	
Anti-epilepsy drug						0.17
No	46(38.98%)	35(29.66%)	16(13.56%)	3(2.54%)	100(84.75%)	
Yes	6(5.08%)	9(7.63%)	1(0.85%)	2(1.69%)	18(15.25%)	
EIAED						0.63
No	51(43.97%)	42(36.21%)	17(14.66%)	5(4.31%)	115(99.14%)	
Yes	0(0%)	1(0.86%)	0(0%)	0(0%)	1(0.86%)	
Heart disease						0.94
No	54(43.20%)	45(36.00%)	16(12.80%)	5(4.00%)	120(96.00%)	
Yes	2(1.60%)	2(1.60%)	1(0.80%)	0(0%)	5(4.00%)	
Hypertension						0.98
No	40(31.75%)	35(27.78%)	12(9.52%)	4(3.17%)	91(72.22%)	
Yes	16(12.70%)	13(10.32%)	5(3.97%)	1(0.79%)	35(27.78%)	
Diabetes						0.68
No	51(40.48%)	41(32.54%)	15(11.90%)	5(3.97%)	112(88.89%)	
Yes	5(3.97%)	7(5.56%)	2(1.59%)	0(0%)	14(11.11%)	
Trauma history						0.63
No	53(42.40%)	47(37.60%)	17(13.60%)	4(3.20%)	121(96.80%)	
Yes	3(2.40%)	1(0.80%)	0(0%)	0(0%)	4(3.20%)	
Family history of malignancy						0.61
No	45(35.71%)	41(32.54%)	15(11.90%)	5(3.97%)	106(84.13%)	
Yes	11(8.73%)	7(5.56%)	2(1.59%)	0(0%)	20(15.87%)	
History of cerebrovascular disease						0.66
No	56(44.80%)	47(37.60%)	17(13.60%)	4(3.20%)	124(99.20%)	
Yes	0(0%)	1(0.80%)	0(0%)	0(0%)	1(0.80%)	
Family history of cerebrovascular disease						0.42
No	49(39.20%)	46(36.80%)	15(12.00%)	4(3.20%)	114(91.20%)	
Yes	7(5.60%)	2(1.60%)	2(1.60%)	0(0%)	11(8.80%)	
Number of lesions						0.83
Multiple	2(1.59%)	1(0.79%)	0(0%)	0(0%)	3(2.38%)	
Single	54(42.86%)	47(37.30%)	17(13.49%)	5(3.97%)	123(97.62%)	
Location of lesion						0.66
Bilateral	0(0%)	2(1.60%)	0(0%)	0(0%)	2(1.60%)	
Left	23(18.40%)	18(14.40%)	7(5.60%)	3(2.40%)	51(40.80%)	
Right	33(26.40%)	27(21.60%)	10(8.00%)	2(1.60%)	72(57.60%)	
Tumor volume (cm^3^)						0.12
Mean ± SD	51.63 ± 41.72	63.22 ± 46.82	50.76 ± 25.89	21.26 ± 12.14	54.68 ± 41.90	
Excision extension						0.61
Gross resection	45(35.71%)	41(32.54%)	15(11.90%)	5(3.97%)	106(84.13%)	
Subtotal resection	11(8.73%)	7(5.56%)	2(1.59%)	0(0%)	20(15.87%)	
IDH1						0.31
Mutant	4(3.45%)	6(5.17%)	4(3.45%)	1(0.86%)	15(12.93%)	
Wildtype	48(41.38%)	37(31.90%)	12(10.34%)	4(3.45%)	101(87.07%)	
IDH2						Not applicable
Wildtype	52(45.22%)	43(37.39%)	16(13.91%)	4(3.48%)	115(100.00%)	
MGMT promoter methylation						0.2
M	32(27.83%)	20(17.39%)	5(4.35%)	3(2.61%)	60(52.17%)	
UM	20(17.39%)	23(20.00%)	10(8.70%)	2(1.74%)	55(47.83%)	
TERT_C228T						0.38
Mutant	20(17.39%)	20(17.39%)	10(8.70%)	3(2.61%)	53(46.09%)	
Wildtype	31(26.96%)	23(20.00%)	6(5.22%)	2(1.74%)	62(53.91%)	
TERT_C250T						0.34
Mutant	12(10.43%)	4(3.48%)	3(2.61%)	1(0.87%)	20(17.39%)	
Wildtype	39(33.91%)	39(33.91%)	13(11.30%)	4(3.48%)	95(82.61%)	
Diagnosis						0.41
CNS4	4(3.15%)	6(4.72%)	4(3.15%)	1(0.79%)	15(11.81%)	
GBM	49(38.58%)	37(29.13%)	12(9.45%)	4(3.15%)	102(80.31%)	
GBM-NOS	3(2.36%)	6(4.72%)	1(0.79%)	0(0%)	10(7.87%)	

EIAED, enzyme inducing anti-epilepsy drug; IDH 1/2, isocitrate dehydrogenase 1/2; MGMT, O6-methylguanine (O6-MeG)-DNA methyltransferase; TERT, telomerase reverse transcriptase; CNS, central nervous system; GBM, glioblastoma; NOS, not otherwise specified.

^*^ p value indicate difference between BMI categories and were calculated by ANOVA analysis or χ² test, as appropriate. P < 0.05 was considered statistically significant.

**Figure 2:**
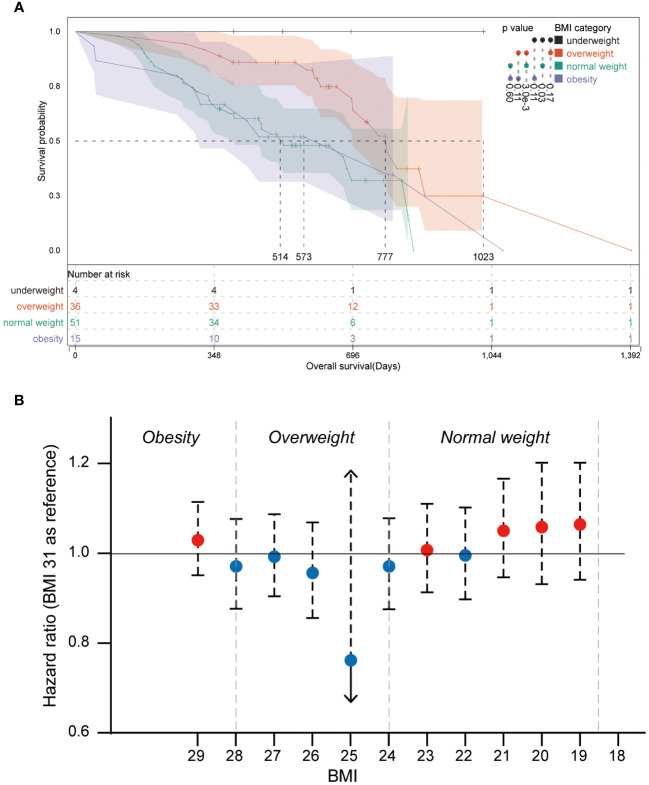
Kaplan-Meier plot and forest plot of the relative hazard ratio of BMI categories **(A)** Kaplan-Meier plot of patients from different BMI categories. Log-rank significances between groups are shown beside the color bar. **(B)** Forest plot of the relative hazard ratio of round-BMI grouped patients, and BMI 31 is set as a reference in the univariate Cox regression.

The survival analysis was performed in the comparison of BMI subsets, and the Kaplan-Meier curve is shown in **Figure 2A**. The median OS was 1023 days in the underweight group, 514 days in the normal weight group, 777 days in the overweight group, and 573 days in the obesity group. Log-rank test showed a significantly higher OS for patients in the overweight group compared to patients in the normal weight group (p = 0.003), and a trend of favoring a better OS of the overweight group compared with the obesity group was observed (p = 0.110). Patients in the underweight group showed superior OS compared with patients in the normal group (p = 0.030); however, the limited sample size of the underweight group makes the findings inconclusive. Therefore, the underweight group has been principally excluded from the analyses of this study. Overweight patients were shown to have superior OS outcomes, but not obese patients. It seems to be a paradox that gaining weight improves OS, up to a certain level.

Further analysis was performed to explore the OS superiority and the breakpoints of OS-beneficial BMI in patients with overweight. Univariate Cox regression was performed for all BMI subgroups using the BMI 31 group as a reference (**Figure 2B**). Although no significant hazard ratios were observed in round-BMI subsets, it was observed that patients of round-BMI 25 and 26 obtained slightly superior OS outcomes with standard-of-care of glioblastoma.

**Figure 3:**
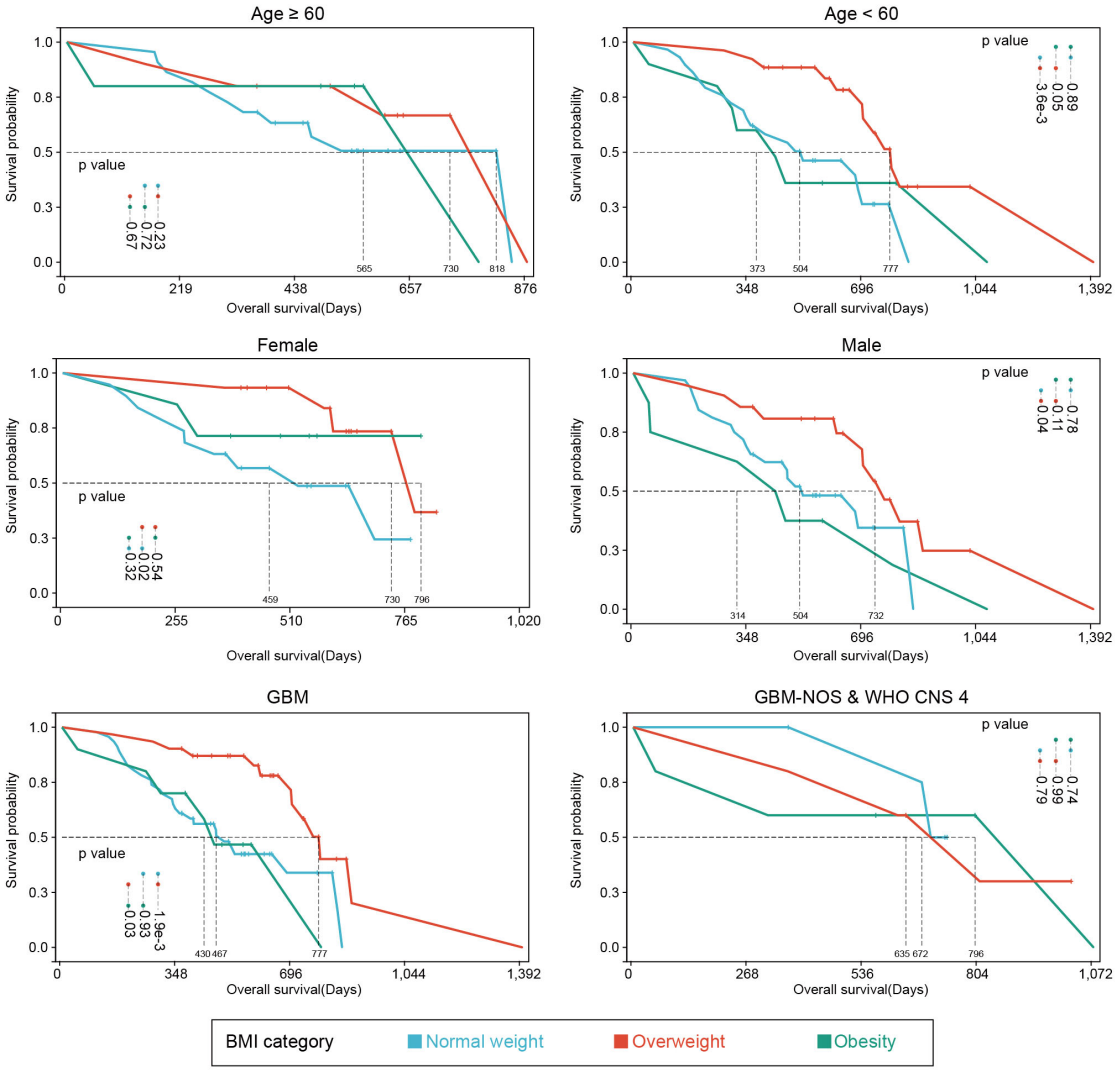
Kaplan-Meier plots of subgroup analyses. Survival analyses of different subgroup settings. Median survival times and significances were shown in the annotations.

**Figure 4:**
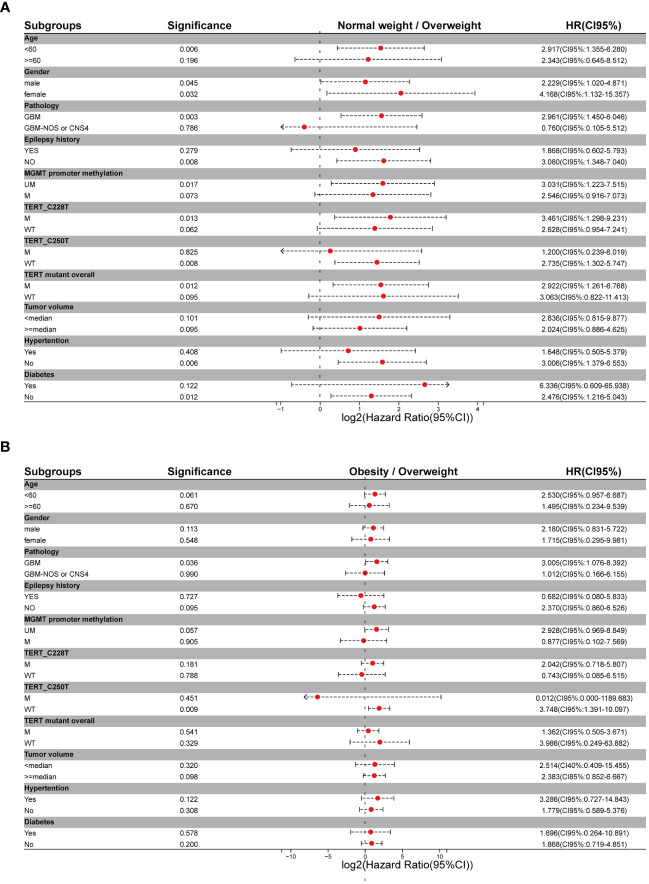
Forest plots of metadata of hazard ratios for different subgrouping settings **(A)** Hazard ratios for comparison of normal weight vs overweight setting. **(B)** Hazard ratios for comparison of obesity vs overweight setting.

For better reliability, sensitivity analysis was also used to explore the OS in BMI clusters (excluding the underweight group) within different subgrouping arrangements, including subgrouping by age, sex, integrated diagnosis of tumor, epilepsy history, MGMT promoter methylation status, TERT promoter mutations, and tumor volume. Kaplan-Meier plots and log-rank analyses are shown in **Figure 3** and **Supplementary Figures 1–3**. A univariate Cox regression model was performed to calculate HRs for subgroups in the comparison of normal weight vs. overweight and obesity vs. overweight. **Figure 4** shows a forest plot of HRs by groups where a superiority in OS of overweight group vs. normal weight group within different subgroups can be observed; a similar trend was observed in the comparison of obesity vs. overweight groups.

Explorations of PFS as a secondary outcome were performed on patients in different BMI groups. The median PFS was 241 days in the underweight group, 282 days in the normal weight group, 351 days in the overweight group, and 286 days in the obesity group (**Supplementary Figure 4**). Log-rank test demonstrated no significant PFS difference among BMI categories (**Supplementary Figure 4**). However, using an identical grouping as that in OS analysis (**Supplementary Figures 5-10**) revealed significant PFS differences between the overweight and obesity group in female patients (p=0.010), patients with methylated MGMT promoter (p=0.030), patients without TERT_C228T promoter mutation (p=0.020), patients without TERT_C250T promoter mutation (p=0.0095) and patients without diabetes (p=0.040).

## Discussion

The index of weight relation to height (BMI) has been used in many studies to assess the risk of death. Standard BMI categories were developed by the World Health Organization (WHO) and the National Heart, Lung, and Blood Institute (NHLBI), namely underweight (<18.5), normal weight (>=18.5, <24.9), overweight (>=25, <29), and obesity (>30) (16). However, non-routine categorization of BMI was conducted in several studies. It is worth noting that the diversity of body compositions, age, and race observed in patients with identical BMI led to disparate clinical outcomes (10). It is acknowledged that overweight and obesity condition increase the morbidity and mortality of coronary disease, hemorrhagic stroke, and ischemic stroke in the Asia-Pacific region (17), which is supported by large-scale epidemiological studies of wide populations (18). However, several studies have challenged this hypothesis by demonstrating that overweight and early obese status are associated with improved survival in patients suffering from various cancerous diseases, such as colorectal and renal cancers, colorectal metastases, acute myeloid leukemia, and lymphoma (19). This finding is termed the “obesity paradox”, which occurs when the risk of clinical outcome is significantly reduced for BMI values above 22.5 kg/m^2^. For patients with very high BMI, risk either returns to unity or is increased in cancer populations (20). Mortality to BMI curves is commonly U-shaped (increased mortality at both ends) (10).

Studies have analyzed the obesity paradox in various diseases, and it has been well described in studies of cardiovascular diseases (21, 22). The association between improved survival and obesity, described by measurement of adiposity from multiple aspects, in heart failure patients has been reported (23). A recent study demonstrated that the short and long-term outcomes tend to improve in patients with obesity undergoing surgical procedures compared to normal-weight individuals (11). Though the reliability of an accurate measurement of adiposity has been questioned, BMI is a convenient parameter in describing obesity-paradox and predicting clinical outcomes in various diseases. For hospitalized and ICU patients or those with chronic illnesses, a J-shaped relationship between BMI and mortality has been demonstrated, with overweight and moderate obesity being protective compared with a normal BMI or more severe obesity (24). In addition, BMI has been used to evaluate prognosis in numerous cancers (19, 25, 26). Among patients with cancer, such as lung cancer, colorectal cancer, renal cell carcinoma, and diffuse large B-cell lymphoma, higher BMI is associated with improved survival compared with normal-weight patients (25, 27).

The vast majority (>75%) of gliomas are high-grade (WHO grade 3 and 4), with the most common and aggressive form of glioma being GBM; WHO grade 4 gliomas, particularly GBM can exhibit pronounced intra-tumoral heterogeneity that confounds clinical diagnosis and management, with a dismal prognosis (28). Despite the increasing emergence of novel genetic and epigenetic biomarkers, it is still challenging to predict clinical outcomes of gliomas or to guide individualized therapy (29–33). BMI as a rapidly and easily acquired parameter in clinical scenarios shows potential capability as a prognostic factor in glioma. A recent study accomplished by Chambless et al. argued that obesity is an independent risk factor for poor outcomes in patients with high-grade glioma, and elevated BMI should be considered when stratifying risk for patients with high-grade glioma (34). However, the pooled analysis of five studies demonstrated a decreased OS in patients with lower BMI compared with patients with obesity (35). Consequently, the prognostic value of BMI in patients with GBM was proposed in several studies (12–14). The unavailability of MGMT promoter methylation status may be a limitation for these studies, which can affect the sensitivity of patients to temozolomide chemotherapy and the OS of patients. In addition, different studies use different BMI categories. Currently, based on the 2021 WHO classification of CNS tumors, no consensus has been achieved about obesity-paradox from the aspect of BMI in primary WHO grade 4 gliomas.

Our study revealed that survival risk showed a U-shape regression within escalating BMI (**Figure 2**), in which round-BMI grouping of the cohort is used for Cox regression analysis. Several clues may help to understand the paradox that low weight is associated with higher mortality, and patients who have normal weight at the time of diagnosis may have previously been overweight or obese before experiencing unintentional weight loss (25). Weight loss is often recognized as a marker of more aggressive cancer and/or advanced activity, even a marker of subclinical tumor activity that can impact lipid metabolism as early as two years before a diagnosis is made (10). In a large prospective cohort of lung cancer, the percentage of patients experiencing pre-diagnosis weight loss ranged from 35% in patients with obesity to 75% in patients with underweight. The relationship between survival and severity of pre-diagnosis weight loss tended to be linear (36). Primary WHO grade 4 gliomas among patients with overweight and obesity may have less aggressive characteristics compared with those among normal-weight patients (19). Molecular heterogeneity is a major characteristic of tumors, and obesity is associated with more indolent molecular variants, including reduced fatty acid synthase (FASN) expression (19). The survival advantage originating from higher BMI may be associated with differences in fatty acid metabolism, and the statistical significance in both FASN and the immediate upstream enzyme acetyl-CoA carboxylase (ACACA) and its encoded protein ACC were observed in patients with obesity and patients with normal weight. FASN is downregulated in patients with obesity but upregulated in patients with normal weight, and the overexpression of FASN is associated with aggressive disease and poor prognosis in several cancer types, including renal cell carcinoma, colon cancer, and prostate cancer (37–40). FASN encodes rate-limiting enzymes involved in fatty acid synthesis, which is a process essential for tumor growth and associated with the incidence of cancer-specific death (37). Patients with overweight and obesity may show superior treatment response and better tolerance for adjuvant chemotherapy compared with normal-weight patients, which might be the consequence of differential pharmacokinetics of cancer treatment regimens (19, 41). Even high BMI appears to be independently associated with improved survival with immune checkpoint inhibitor therapy (atezolizumab) in patients with non-small cell lung cancer (NSCLC), and a linear association between increasing BMI and OS was observed (42). Excess adipose tissue may have a protective role as a nutrient reserve, which helps improve the survival of patients enduring chemoradiotherapy and adjuvant chemotherapy. In addition, overweight people pay more attention to their health status and have more regular medical follow-ups due to their higher risk of comorbidities (36). Therefore, a sequential record of BMI during the process of disease progression would be helpful to further understand the clinical outcome-predicting role of BMI in all fields of research, especially for cancer research, because universal weight loss happens in the majority of cancer patients. Proposing an ideal BMI for cancer patients would be useful in the administration of cancer, which still requires considerable effort.

This study has several limitations. A relatively small size cohort was used in this study, and a larger sample size would certainly be helpful to make the conclusion more solid. Cohorts from multiple regions or races would possibly improve the extrapolation of conclusions. Few patients were finally enrolled in the underweight group so the association between clinical outcome and patients with underweight remains ambiguous. Currently, the sensitivity and reliability of BMI in assessing malnutrition and adiposity have been questioned. Novel nutrition-related parameters, such as waist circumference, waist-to-hip ratio, and skinfold would make BMI more comprehensive in assessing body condition; body composition assessment techniques, such as dual-energy X-ray absorptiometry, computed tomography (CT) and MRI, and biological parameters such as serum albumin concentration could also be considered.

## Conclusions

We conducted a retrospective real-world study to assess the prognostic capability of BMI in patients with primary WHO grade 4 gliomas. BMI was shown to be a feasible prognostic factor. Overweight patients were shown to have superior survival benefits. While the limited sample size of this study may impact the reliability of the results, ideal weight management may increase the survival benefits of patients. Future studies will be required to consider novel nutritional parameters and the history of weight change to develop a comprehensive prognosis of patients with primary WHO grade 4 gliomas. Finally, a multicenter study with a larger sample size may provide further evidence to support the “obesity paradox” in WHO grade 4 gliomas.

## Data availability statement

The original contributions presented in the study are included in the article/**Supplementary Material**. Further inquiries can be directed to the corresponding author.

## Ethics statement

The studies involving humans were approved by the Ethics Committee of Beijing Tiantan hospital (JS2012-001-03). The studies were conducted in accordance with the local legislation and institutional requirements. Written informed consent for participation was not required from the participants or the participants’ legal guardians/next of kin in accordance with the national legislation and institutional requirements. Written informed consent was obtained from the individual(s) for the publication of any potentially identifiable images or data included in this article.

## Author contributions

JW: Writing – review & editing, Writing – original draft, Formal analysis. ZH: Writing – review & editing, Writing – original draft, Formal analysis. RL: Writing – review & editing, Validation, Methodology, Formal analysis. WW: Writing – review & editing, Supervision, Resources, Formal analysis. NH: Writing – review & editing, Validation, Supervision, Resources. KZ: Writing – review & editing, Supervision, Resources. SH: Writing – review & editing, Supervision, Project administration, Methodology. JF: Writing – review & editing, Validation, Supervision, Formal analysis. JC: Writing – review & editing, Validation, Supervision. NJ: Writing – review & editing, Funding acquisition, Conceptualization.
