# Supplementary material for: Association of body mass index with clinical outcome of primary WHO grade 4 glioma
Source: Front Oncol. 2024 Apr 29;14:1318785. doi: 10.3389/fonc.2024.1318785 (PMC11089228; doi:10.3389/fonc.2024.1318785)
Supplement: Supplementary file 1 [file DataSheet_1.docx]

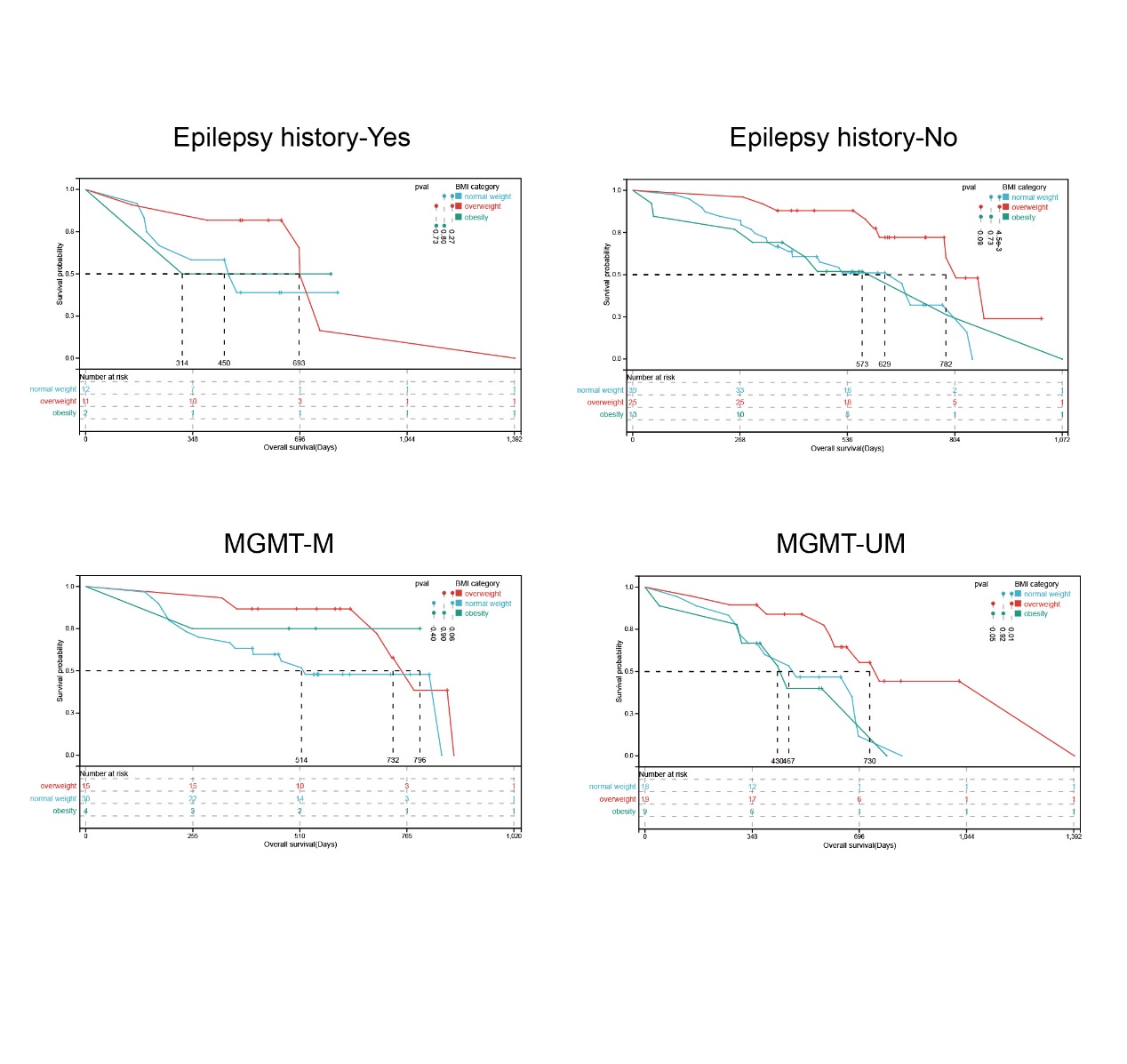


**Supplementary Fig1** Kaplan-Meier plots of subgroup analyses. Survival analyses of different subgroup settings. Median survival times and significances were shown in the annotations.


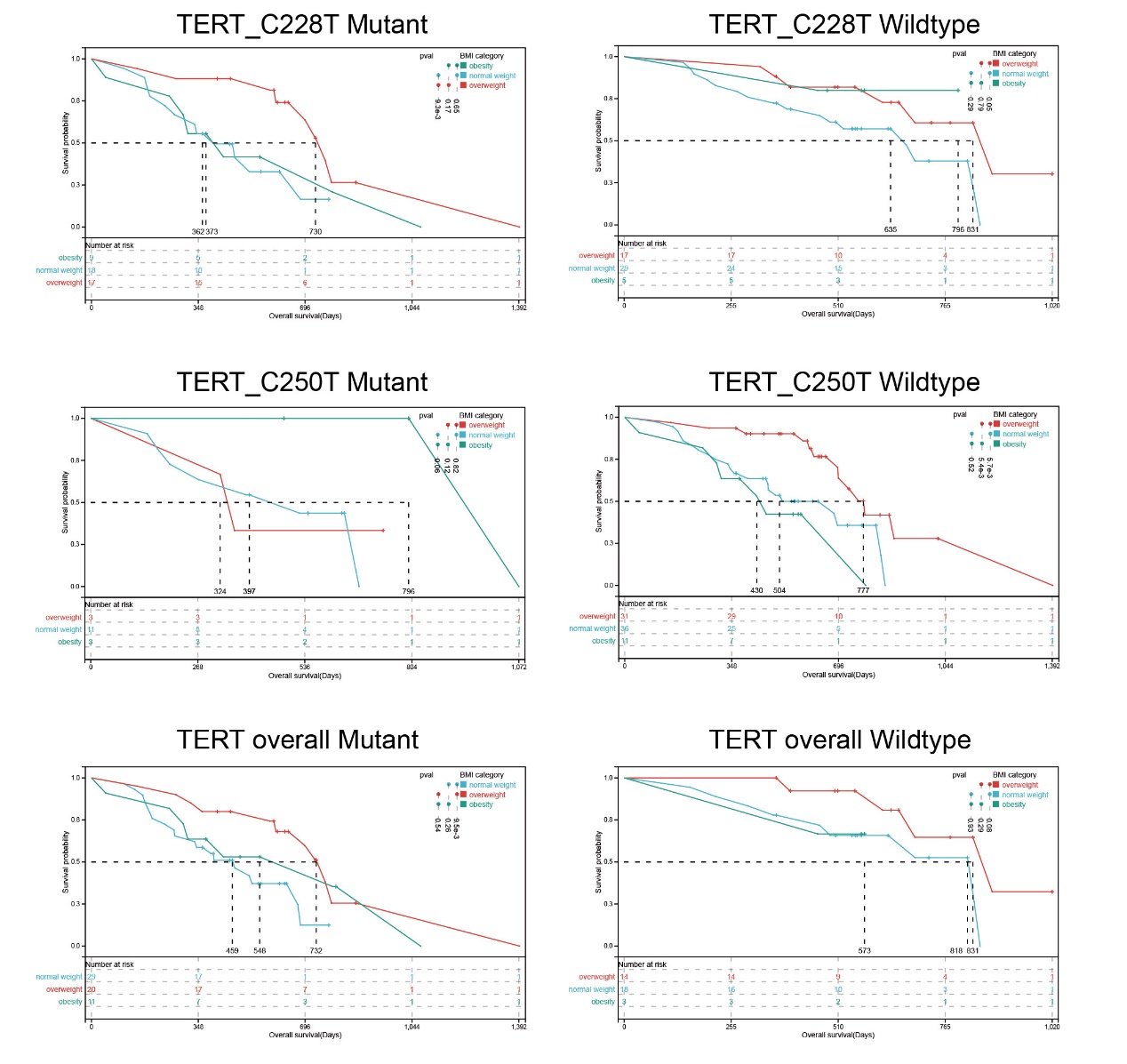


**Supplementary Fig2** Kaplan-Meier plots of subgroup analyses. Survival analyses of different subgroup settings. Median survival times and significances were shown in the annotations.


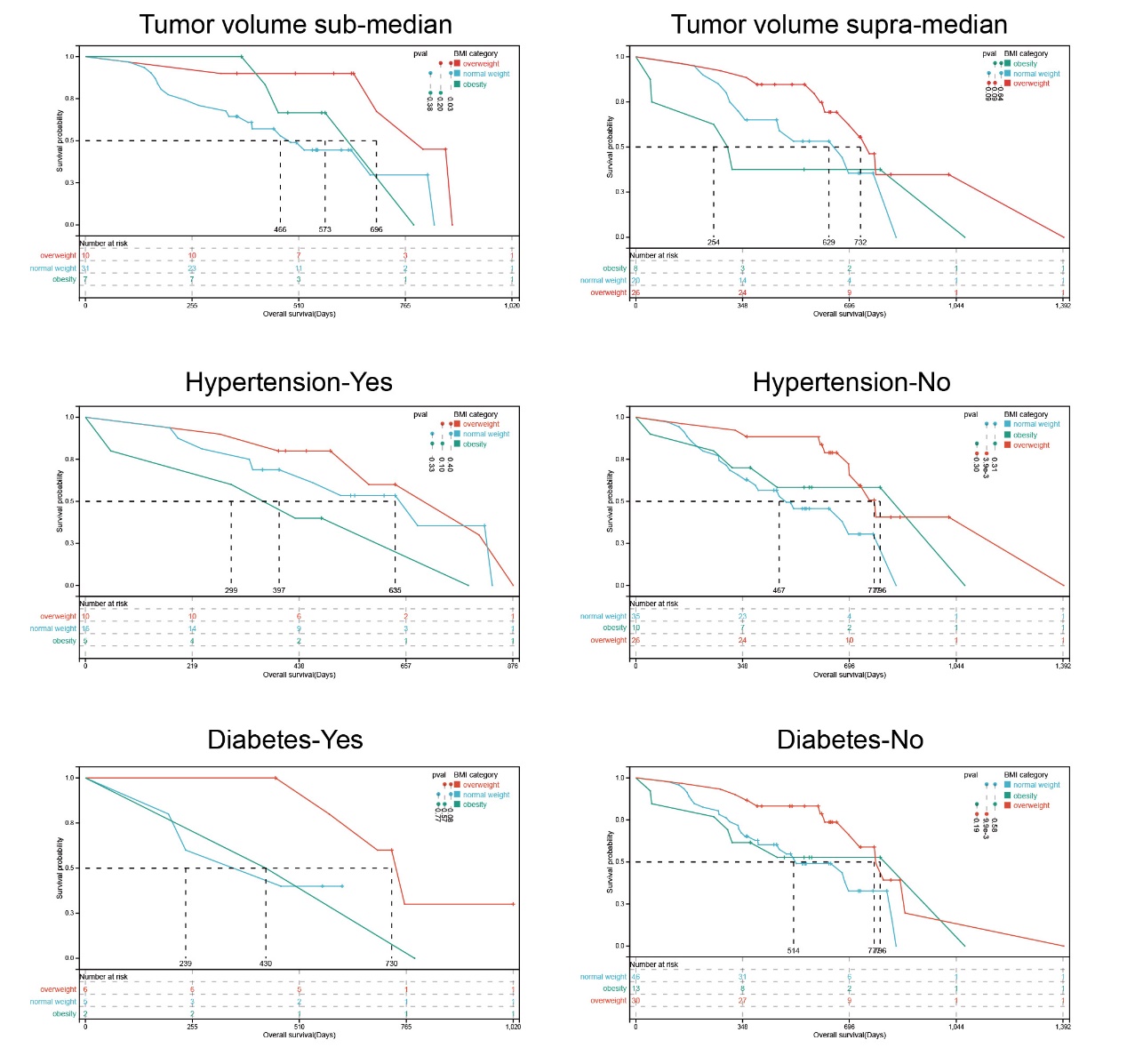


**Supplementary Fig3** Kaplan-Meier plots of subgroup analyses. Survival analyses of different subgroup settings. Median survival times and significances were shown in the annotations.


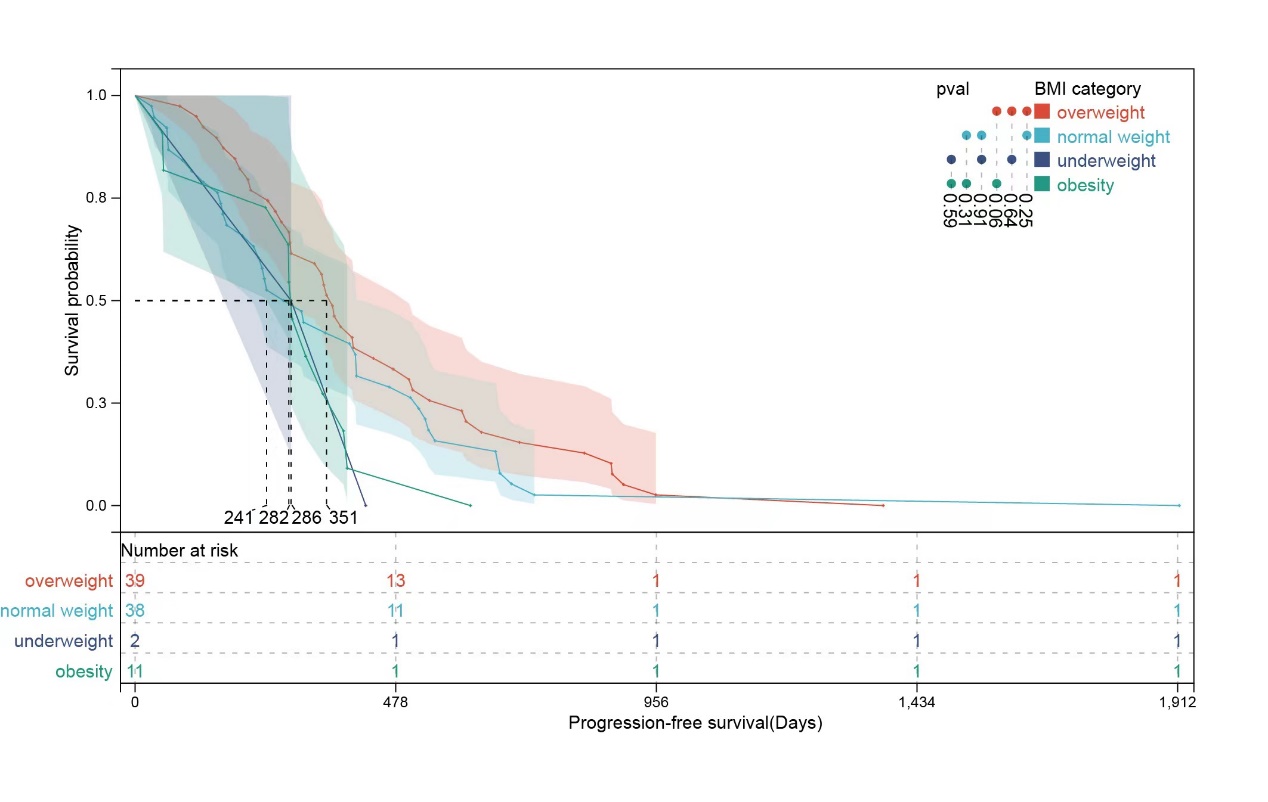


**Supplementary Fig4** Kaplan-Meier plot (PFS as outcome) of BMI categories. Median survival times and significances were shown in the annotations.


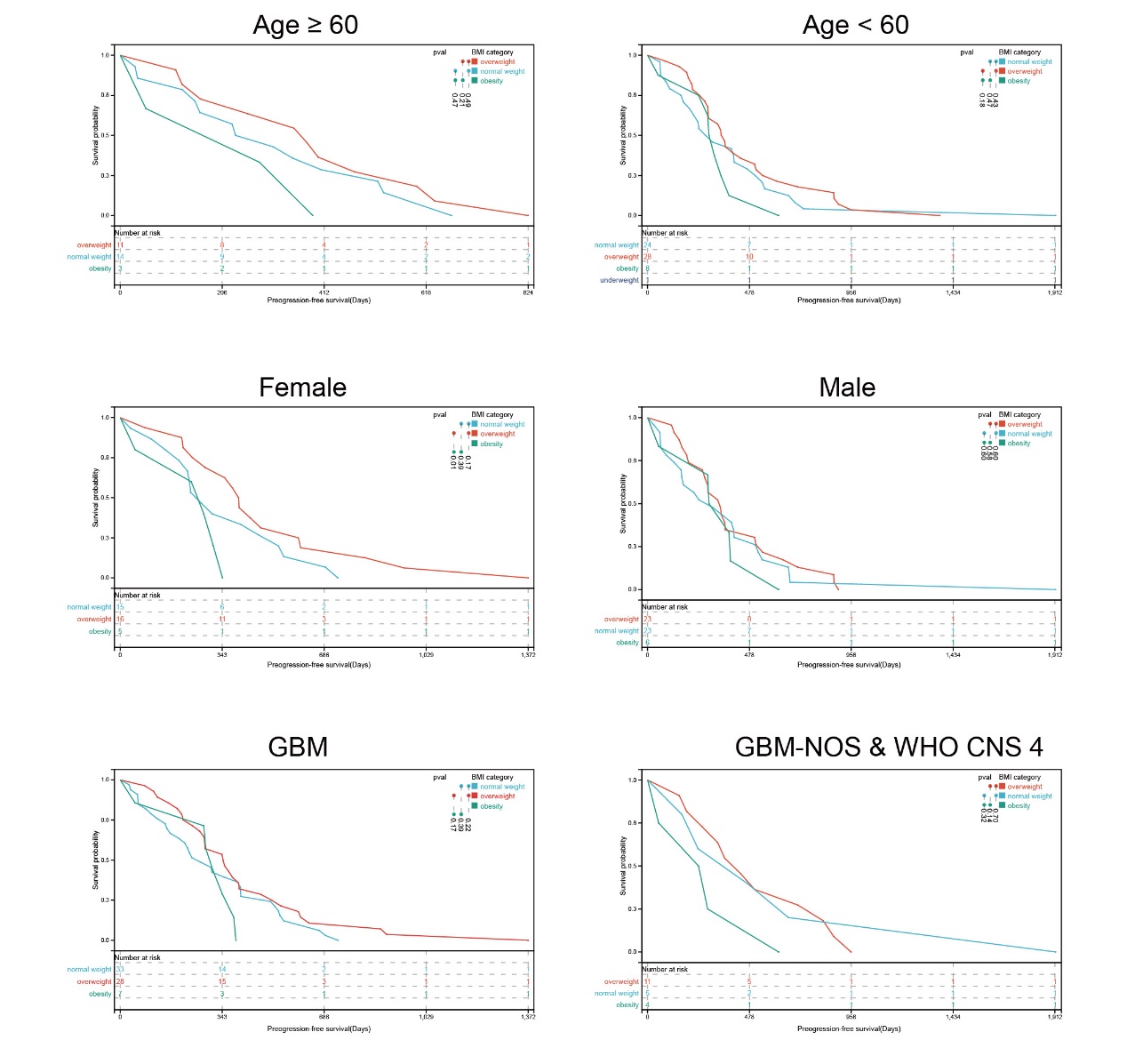


**Supplementary Fig5** Kaplan-Meier plots of subgroup analyses. Survival analyses of different subgroup settings (PFS as outcome). Median survival times and significances were shown in the annotations.


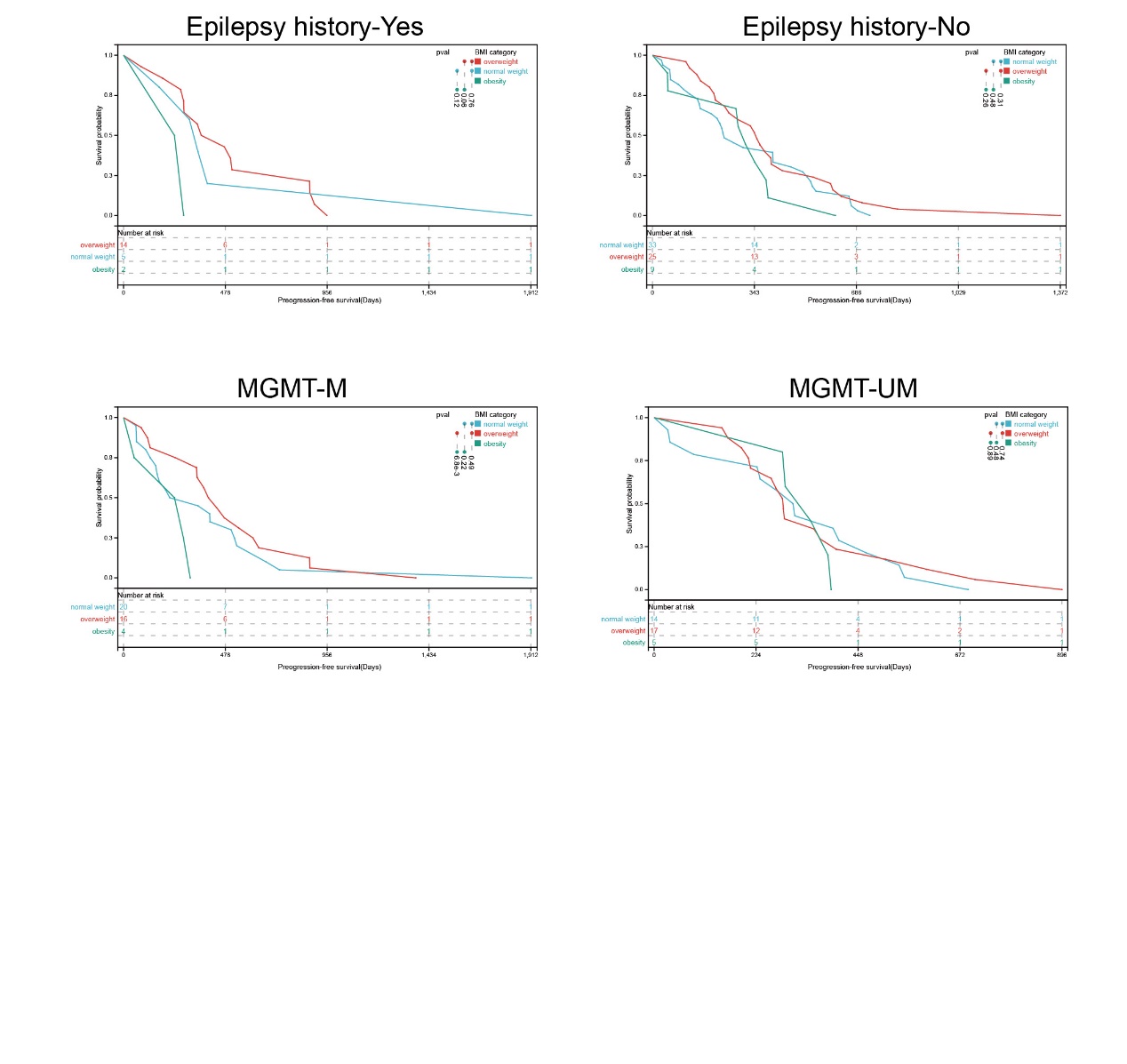


**Supplementary Fig6** Kaplan-Meier plots of subgroup analyses. Survival analyses of different subgroup settings (PFS as outcome). Median survival times and significances were shown in the annotations.


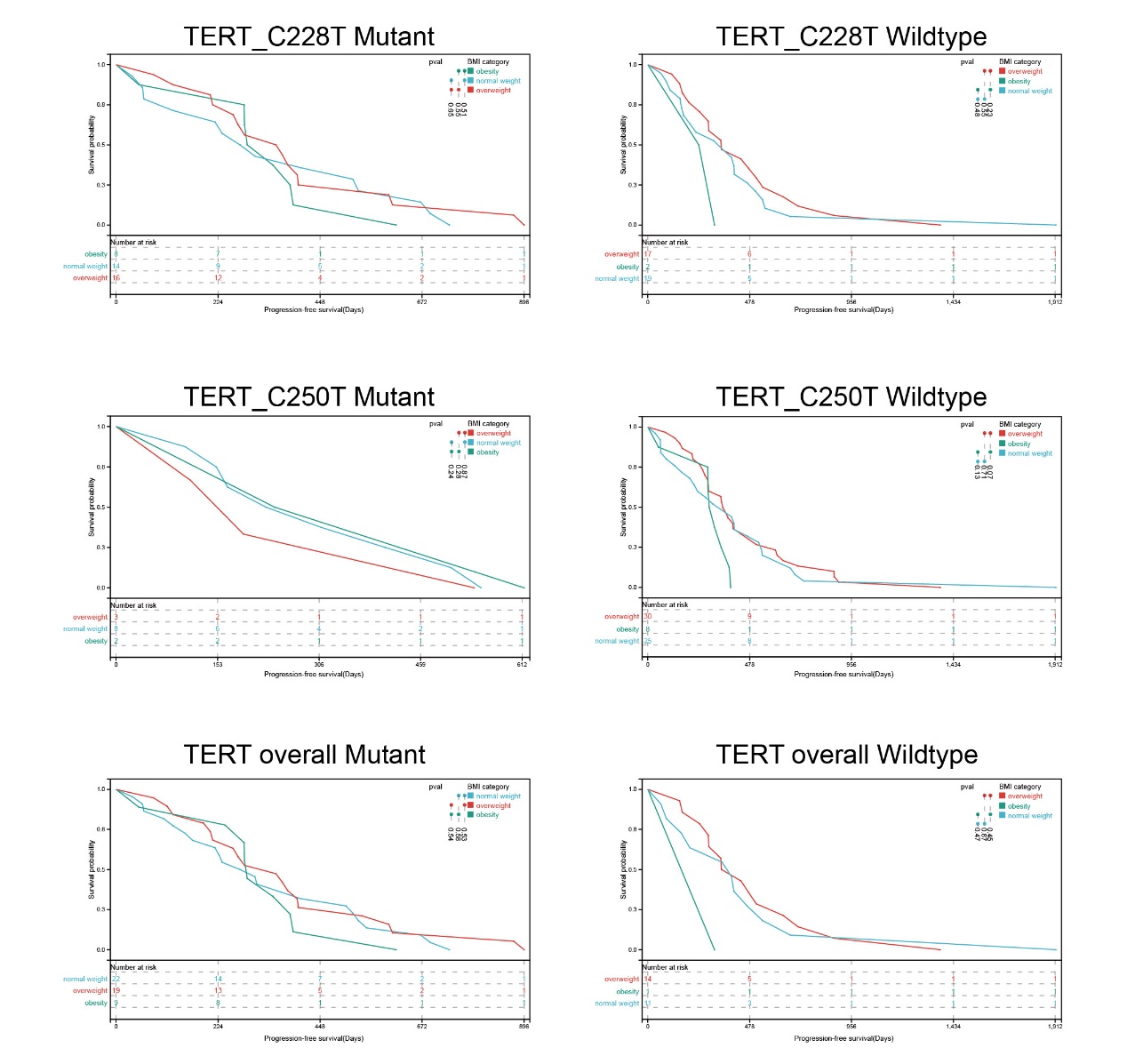


**Supplementary Fig7** Kaplan-Meier plots of subgroup analyses. Survival analyses of different subgroup settings (PFS as outcome). Median survival times and significances were shown in the annotations.


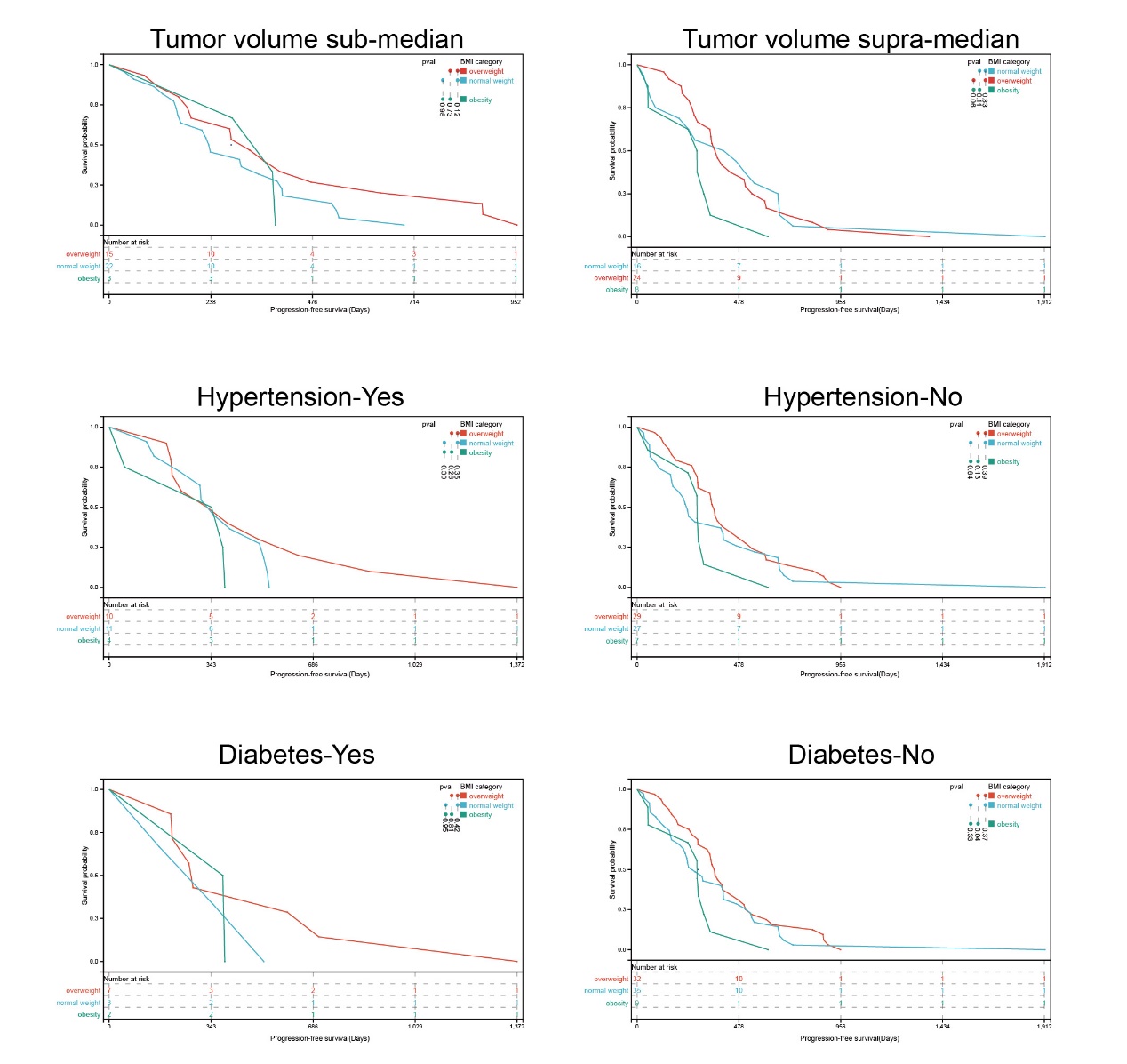


**Supplementary Fig8** Kaplan-Meier plots of subgroup analyses. Survival analyses of different subgroup settings (PFS as outcome). Median survival times and significances were shown in the annotations.


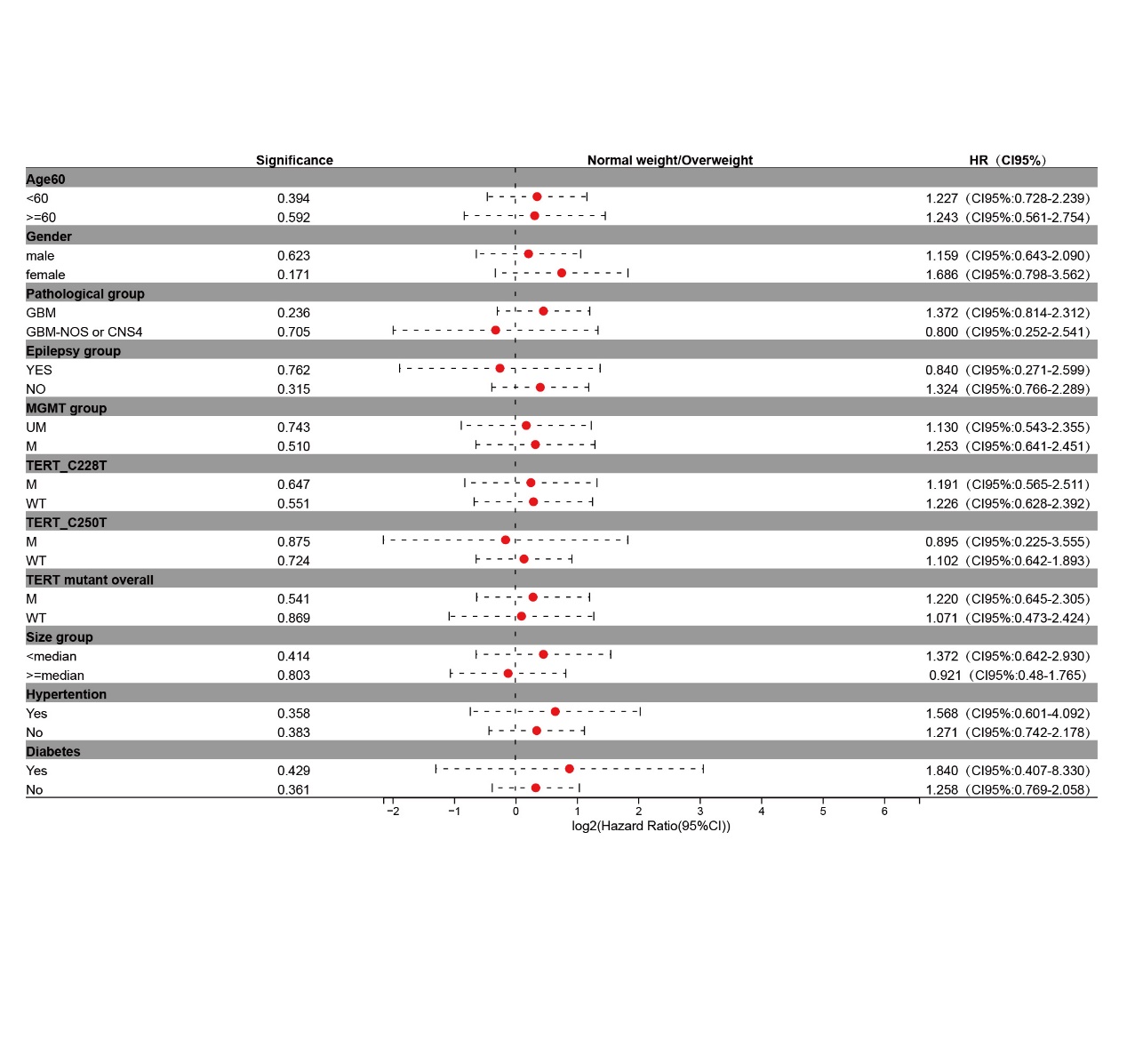


**Supplementary Fig9** Forest plot of meta data of hazard ratios for different subgrouping setting. Hazard ratios for comparison of normal weight vs overweight setting.


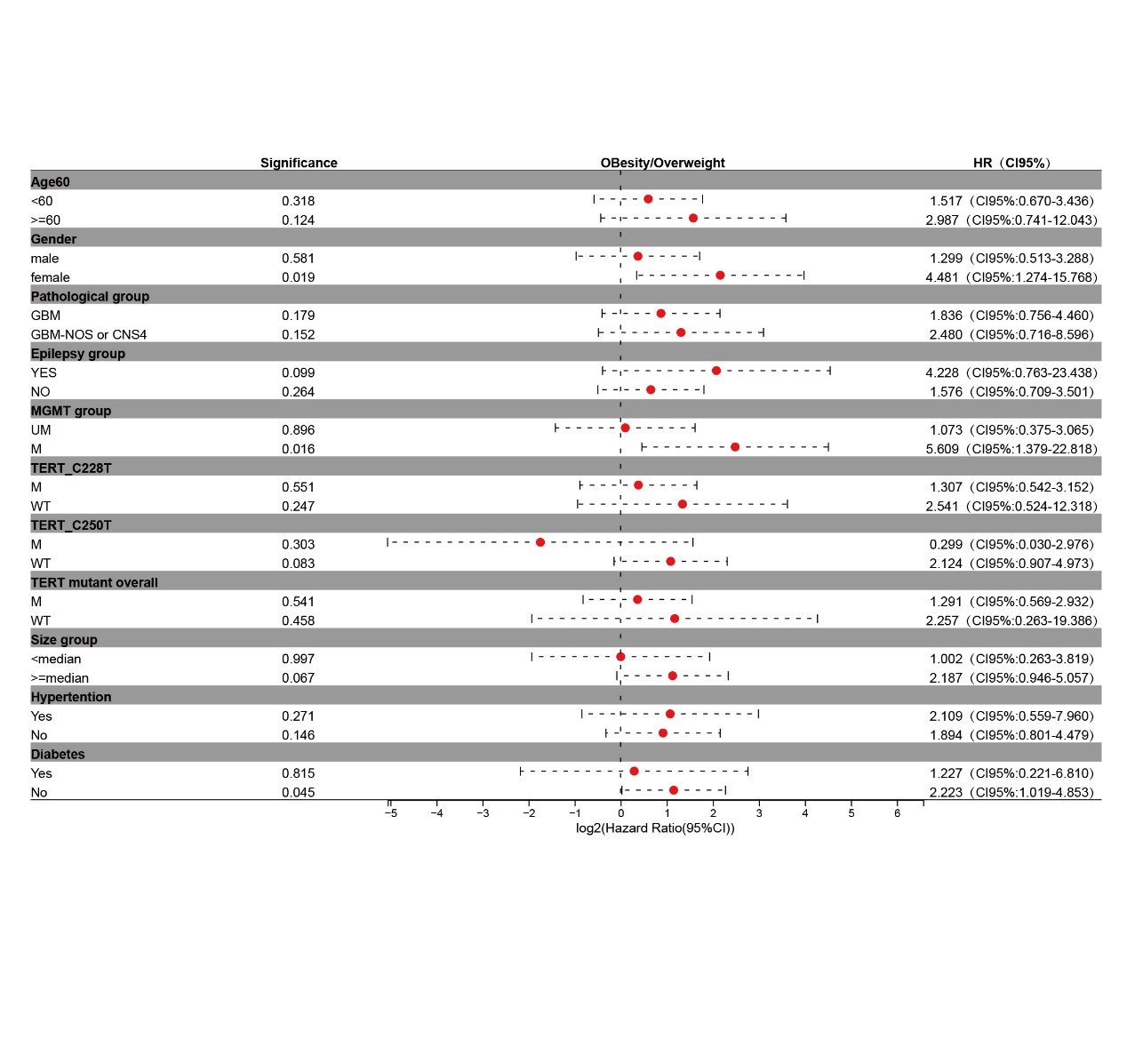


**Supplementary Fig10** Forest plot of meta data of hazard ratios for different subgrouping setting. Hazard ratios for comparison of obesity vs overweight setting.
